# Thermographic Imaging in Cultural Heritage: A Short Review

**DOI:** 10.3390/s22239076

**Published:** 2022-11-23

**Authors:** Vasiliki Dritsa, Noemi Orazi, Yuan Yao, Stefano Paoloni, Maria Koui, Stefano Sfarra

**Affiliations:** 1 NDT Laboratory, Department of Materials Science & Engineering, School of Chemical Engineering, National Technical University of Athens, Iroon Polytechniou No. 9, Zografou Campus, 15780 Athens, Greece; 2 Dipartimento di Ingegneria Industriale, Università Degli Studi di Roma Tor Vergata, Via Del Politecnico 1, 00133 Rome, Italy; 3 Department of Chemical Engineering, National Tsing Hua University, Hsinchu 30013, Taiwan; 4 Department of Industrial and Information Engineering and Economics (DIIIE), University of L’Aquila, Monteluco di Roio, 67100 L’Aquila, Italy

**Keywords:** pulsed infrared thermography, ground penetrating radar, cultural heritage investigation, non-destructive evaluation, documentary materials, panel paintings, mosaics

## Abstract

Over the recent period, there has been an increasing interest in the use of pulsed infrared thermography (PT) for the non-destructive evaluation of Cultural Heritage (CH). Unlike other techniques that are commonly employed in the same field, PT enables the depth-resolved detection of different kinds of subsurface features, thus providing helpful information for both scholars and restorers. Due to this reason, several research activities are currently underway to further improve the PT effectiveness. In this manuscript, the specific use of PT for the analysis of three different types of CH, namely documentary materials, panel paintings–marquetery, and mosaics, will be reviewed. In the latter case, i.e., mosaics, passive thermography combined with ground penetrating radar (GPR) and digital microscopy (DM) have also been deepened, considering their suitability in the open field. Such items have been selected because they are characterized by quite distinct physical and structural properties and, therefore, different PT (and, in some cases, verification) approaches have been employed for their investigations.

## 1. Introduction

Scientific investigations of Cultural Heritage (CH) are of relevant importance since they allow for the gathering of valuable information, such as the ones concerning their manufacturing processes and/or preservation conditions. Among other techniques, Pulsed Infrared Thermography (PT) is nowadays established as an effective tool thanks to its remote character allowing the in situ non-destructive investigation of the artworks by means of relatively simple experimental procedures [1].

PT falls in the active modality. Contrary to passive thermography, active thermography requires an external heat source to stimulate the materials under test. Commonly, halogen lamps, high-power photographic flash, and laser beams are widely used, and other high-power cinematographic lamps and quartz line infrared (IR) lamps are used. In addition, active thermography is subdivided into lock-in thermography (LIT), PT, step-heated thermography (SHT), and vibrothermography (VT) according to external heating methods, and PT and LIT are the most utilized. Parker and other researchers integrated the PT idea into various non-destructive testing (NDT) applications. The concept of a PT system for defect detection consists of applying a short time and powerful energy pulse to an object and then recording the temperature rise, decay, or both curves in transient mode.

PT is one of the active infrared techniques, which uses an optical device as an external heat source. Among the active thermography techniques described above, it is the easiest to apply and widely used [2,3,4,5,6,7,8,9]. From the physics point of view, PT relies on the spatially resolved detection of the transient IR emission from the sample surface (in both the mid-wave (MW) and the long-wave (LW) IR spectral ranges), typically induced by the absorption of short light pulses.

To this aim, an infrared camera is employed to record a sequence of images, referred to as thermograms, each corresponding to a different delay time with respect to the onset of the heating pulse. One of the peculiar PT abilities is given by the possibility to perform depth-resolved characterizations of the subsurface features lying in the sample, thus providing important information for scholars and restorers. In fact, after the initial heating, the induced temperature increase diffuses into the sample over a depth that increases with time [10,11,12]. Local subsurface inhomogeneities in the sample’s physical properties can introduce significant modifications to such a diffusion process, thus leading to corresponding variations in the amount of IR radiation emitted from the above surface area. Consequently, such subsurface elements can be detected through the contrasting features in the thermograms recorded at a delay time progressively increasing with the feature position depth [13,14,15].

Although PT has already been successfully applied to the analysis of different kinds of artworks, such as bronze artifacts [16] or mural paintings/frescoes [17], to name a few, several research activities are currently underway to improve the PT effectiveness. It must be considered that in the investigation of artworks, the amount of heating energy must be limited in order to prevent damage caused by a too-large temperature increase or by excessive light absorption.

However, such a limitation results in recorded images characterized by poor signal-to-noise-ratio (SNR) conditions. Owing to this reason, several efforts are currently devoted to the development of image processing tools in order to enhance the visibility of the detected features concealed in the noise level [18,19]. In this respect, the development of high-quality thermal cameras and related equipment also plays a crucial role [20,21]. In addition, integrated approaches where PT is employed in combination with other techniques, able to provide complementary results, are attracting increasing attention because of the possibility of obtaining more accurate investigations [22,23].

In the following, the specific application of PT to the analysis of three different types of CH artifacts will be reviewed. Initially, the use of PT for the detection of graphical features in documentary materials is described. Here, the contrast in the thermograms mainly originated from the different optical properties of the inks with respect to those of the surrounding materials. Due to this reason, in this kind of study, PT is often employed in combination with IR reflectography because of its more significant sensitivity in comparison with that of PT for features located close to the surface and, consequently, of the possibility of obtaining complementary results.

After that, the authors will report on the inspections of panel paintings where, as mentioned before, the thermograms are often characterized by poor SNR conditions due to the limited amount of heating energy. In this regard, image processing tools that have been developed accurately so as to enable the visualization of otherwise undetectable features will be presented.

Finally, the diagnosis of mosaics will be described. In these studies, passive thermography has also been employed because of the possibility of inspecting large sample areas thanks to solar energy heating. In addition, the ground penetrating radar (GPR) technique has been adopted in combination with the passive thermography approach so as to probe the mosaics over a more significant depth and, hence, to detect shallow features [24,25,26].

To summarize, the main reasons explaining why the PT or passive infrared thermography (IRT) inspections should be performed by scientists or required by conservators are reported in Table 1. The symbol “*√*” is linked to the benefits of inspections. As it is possible to see, IRT is able to cover a broad spectrum of features by obtaining exciting results [27,28,29,30]. Due to the physical reasons explained above, also related to the nature of the top layer (i.e., thickness, thermal properties, heterogeneous materials, etc.), the MW and the LWIR radiations fail to identify alone the hidden features in some types of artworks; in these cases, the joint use of additional NDTs may be of help [31,32,33]. The same joint approach, but mostly oriented toward the use of chemical analyses, is of support in the identification of art copies, fakes, and forgeries [34]. Since panel paintings are usually characterized by a hidden layer of canvas, in this case, PT works if the texture can be identified clearly [35,36].

## 2. PT and/or Passive IRT Applications

### 2.1. Documentary Materials

Among different applications, PT has been successfully adopted for the non-destructive detection of subsurface features in documentary materials. In the study of ancient books, PT has been applied to the analysis of bookbinding and, more specifically, to the investigation of the adhesion state among their different parts [37] and/or to the detection of the presence of possible damage [38]. Such experiments have often been carried out by means of integrated approaches combining the use of PT with other techniques able to provide complementary results, such as near-infrared reflectography (NIR) [39], and two-dimensional proton nuclear magnetic resonance relaxometry [40,41].

In addition to the analysis of the inhomogeneities in the book structure, PT enables the detection of the presence of buried graphical features, such as hidden texts or underdrawings on illuminations, even if their influence on the heat diffusion and, hence, on the resulting temperature distribution at the sample surface, is negligible. Such a possibility relies on the fact that library and archive items are made of optically semi-transparent materials and, unlike in the optically opaque ones, both the VIS light heating and the subsequent IR emissions take place over a specific sample depth depending on the sample optical properties. Therefore, the contrast in the recorded PT images may also originate from buried features characterized by different visible (VIS) absorption or IR emission properties with respect to that of the surrounding medium.

In this respect, it is worth pointing out that the detection of subsurface inhomogeneities is granted by the following mechanisms. When the position depth of the buried feature is smaller than the penetration depth of the VIS light, then PT contrast is mainly due to different temperature rises induced at the feature as compared to that at the surrounding parts because of the feature VIS absorption properties. On the contrary, deeply buried features may be possibly reached by the diffusing thermal wave, and owing to their different emissivity, a contrasted IR emission may take place.

Thanks to the latter mechanism, PT has proven to be more effective than IR reflectography [39] for the detection of in-depth buried elements, mainly when they are located beneath optically diffusing layers, such as the ones made of paper [42]. The presence of such layers can significantly prevent the IR radiation from traveling undisturbed back and forth from the surface to the subsurface feature and, consequently, the recording of readable reflectographic images.

On the basis of the considerations reported above, it is clear that the PT signal originating from optically semi-transparent materials is not merely proportional to the temperature variation at the sample surface as in the case of optically opaque ones. Therefore, quantitative evaluation in this kind of specimen is less straightforward than the ones carried out in other CH items, such as ancient bronze statues, due to the large number of sample properties involved in the PT signal description. Nonetheless, numerical models for the PT signal originating from features buried in semi-transparent materials have recently been proposed [43], and their development is still underway. In addition, image processing tools such as wavelet transform thermography (WTT) [44] and higher-order statistics thermography (HOST) [45] have effectively been employed even in the investigation of books to improve the visibility of the detected features in the acquired PT images.

**Figure 1:**
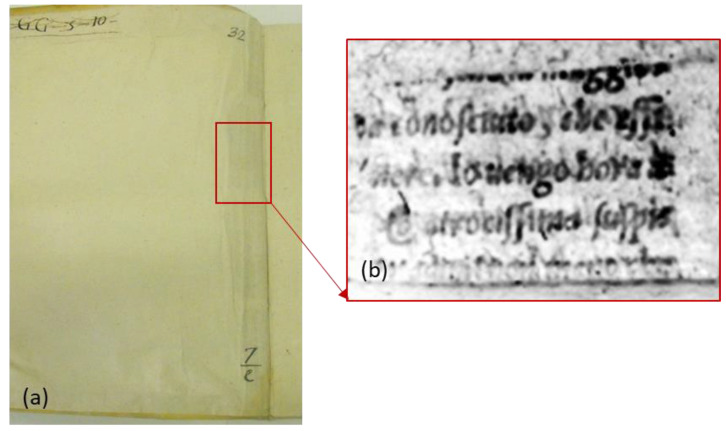
A 17th-century book, *Biblioteca Angelica* (Rome); (**a**) photo of the front end-leaf and (**b**) thermogram corresponding to the area marked by the rectangle in (**a**).

As specifically regards the field dealing with investigations on ancient books, PT has been adopted for the detection of both the earlier scraps that had been reused to reinforce structural parts of the book-bindings and of the written fragments that had been inserted beneath the endpaper to keep the turn-ins well-attached to the board [38,46]. In this respect, it is worth pointing out that, in some cases, the contents of such written scraps can be of great interest to scholars and conservators, being able to provide information on the conservative history of books. For instance, Figure 1 shows the results obtained on the back-end paper of a 17th-century book hosted at the *Biblioteca Angelica* in Rome (Italy) by means of the experimental setup described in Ref. [46]. The thermogram reported in Figure 1b has been recorded right after the VIS heating pulse over the area highlighted by the red rectangle in Figure 1a. As seen, such a thermogram allows the readability of the text on a fragment lying in contact with the back surface of the end leaf. As mentioned before, such a possibility is granted from the localized absorption of the visible heating light at the text ink, which, in turn, produces a more significant local IR emission with respect to the surroundings. Moreover, the IR radiation over the mid-wave infrared (MWIR) has been specifically detected since it is not substantially scattered when propagating through the end leaf on its way to the IR camera, thus allowing a more precise visualization of the text content in the recorded thermogram.

In this regard, it is worth mentioning that similar kinds of subsurface features have been investigated by the mobile macro-*X*-ray fluorescence (XRF) scanner technique that also enabled the mapping of the elemental composition of even the fragments located deeper in the bookbinding. However, the application of this technique is strongly limited to texts made of iron-based inks, thus excluding all carbon-based ones [47].

**Figure 2:**
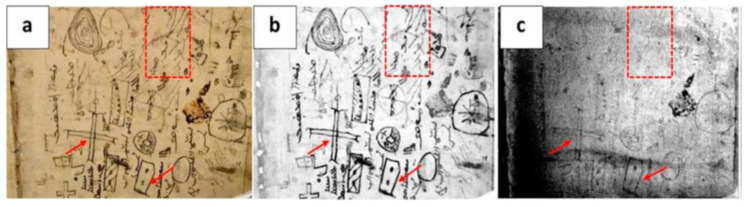
*Libro Sacro of the Church Vergine Maria di Qaeaqosh* o *Baghdide*; (**a**) photograph of the page containing a different kind of inks; (**b**) thermogram, and (**c**) MWIR reflectogram. The arrows indicate the inks containing carbon-based components, while the dashed rectangle marks writing are not more visible, thus revealing the absence of carbon in its composition. (Reprinted with permission from Ref. [38]: *Mid-wave Infrared Reflectography and Thermography for the Study of Ancient Books: A Review, Studies in Conservation*, N. Orazi, copyright 2020 © The International Institute for Conservation of Historic and Artistic Works, reprinted by permission of Taylor & Francis Ltd (Milton Park, Oxfordshire, UK), http://www.tandfonline.com on behalf of The International Institute for Conservation of Historic and Artistic Works).

In the study of original manuscripts, the analysis of the deterioration phenomena which may cause the loss of readability of written parts is of crucial importance, as such investigations are often carried out by means of multispectral and hyperspectral imaging techniques [48,49]. The possibility of discriminating between inks characterized by different compositions may also play a crucial role. In this respect, MWIR reflectography (MIR) has been proven to be an effective tool for the detection of carbon-based inks, since, unlike the metal-based ones, they lead to contrasted reflectographic images obtained in both the short wave infrared (SWIR) and MWIR ranges [50], thus enabling to distinguish between purely metal-based inks and those containing carbon. As an example, Figure 2 shows the image obtained on a page of the *Libro Sacro* of the *Church Vergine Maria di Qaeaqosh* o *Baghdide*. As seen in the photograph of Figure 2a, the investigated page has been written using different kinds of ink, probably applied at other times. The results obtained by PT and MIR are depicted in Figure 2b,c, respectively. In particular, the VIS light absorption at all the inks then makes visible all the writings in the thermogram. On the other hand, in the MIR image, only the writings containing carbon-based components can be observed (such as the ones indicated by the arrows in Figure 2b,c) since they probably absorb the MWIR radiation in a more efficient way than the other ones on the surrounding parchment. On the other hand, writings that do not contain carbon-based components, such as those marked by the dashed rectangles in Figure 2, are no longer visible in the reflectogram of Figure 2c.

### 2.2. Panel Paintings and Marqueteries

Owing to its relatively low heat exposure, high signal-to-noise ratio (SNR), and capability of identifying subsurface features of materials, PT has also been adopted to explore the multi-layer structures of panel paintings. The work of [51] is likely the earliest piece of literature discussing the feasibility of using thermography to detect subsurface defects in panel paintings, which states that “by using short pulses of infrared radiation on the order of tenths of a second in duration, harmless but detectible temperature variations over voids might be created”. Blessley et al. [52] provided comprehensive discussions on the effectiveness, limitations, parameters, and potential risks of applying PT to artworks, including panel painting, providing guidelines for implementing this technique. The authors applied a ∆*T* of 3 ± 0.1 °C measured at the object surface via a k-type thermocouple directly after the pulse. Such a temperature increase with respect to the initial object temperature did not damage the painting under test, and it was considered the maximum ∆*T* allowed. This precautionary value changes case by case; e.g., in [53], using halogen lamps, a ∆*T* of 17 °C was used without seeing—in the course of time—evident signs of pigment degradation/discoloration.

**Figure 3:**
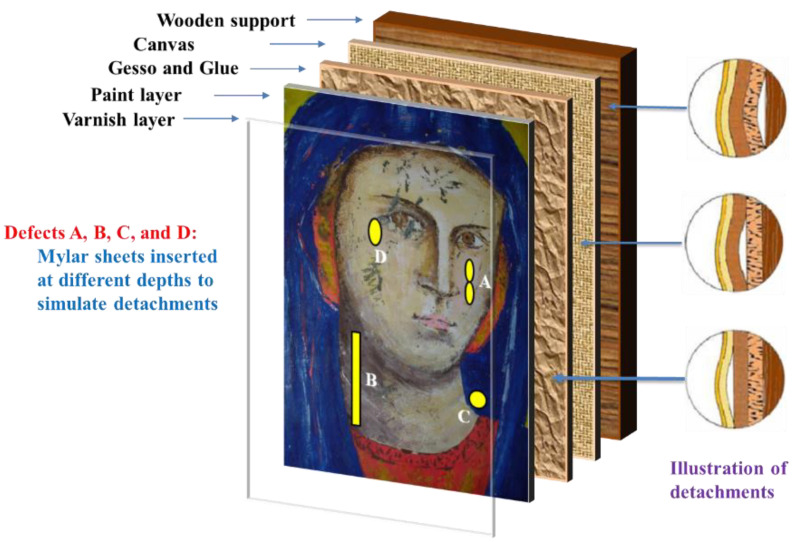
Structure of *Madonna* panel painting. The Figure was redrawn considering Ref. [55].

**Figure 4:**
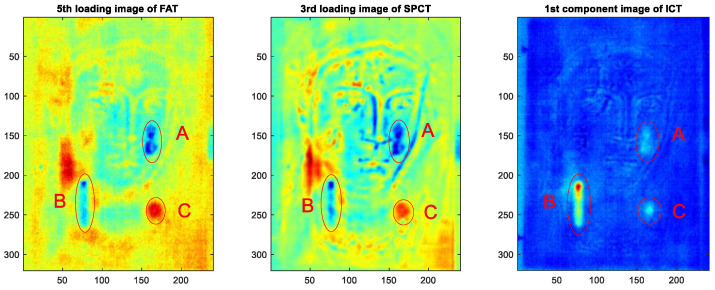
Thermographic data analysis results obtained in *Madonna* panel painting. The authors made these illustrative figures specifically for this manuscript based on the existing dataset and methods proposed in Refs. [4,55].

Although the value of a panel painting is mainly because of its painted surface, i.e., the image layer, the durability of the wooden support is critical to the preservation of the paint. Different factors may cause defects and damage to wooden structures, such as the quality of the selected wood materials, biological infestations, environmental conditions, as well as the conservation and acquisition history of the panel painting. In recent years, a number of post-processing methods have been proposed to enhance the visibility of defects in thermograms obtained from PT experiments on panel paintings. Ibarra-Castanedo et al. [54] implemented differential absolute contrast (DAC) and pulsed phase thermography (PPT) in the investigation of a *Madonna* panel painting manufactured following the procedure of the Renaissance art master *Cennino Cennini* (1370–1427). Figure 3 shows the structure of the specimen, which contains five layers of different materials and functions, as well as four fabricated defects to detect. Later, Yao et al. [55] proposed to use a pixelwise algorithm for time-derivative of temperature (PATDT), sparse principal component thermography (SPCT), and independent component thermography (ICT) to explore the defect information of the same specimen, which further separated the defect signals from the backgrounds. Among these methods, DAC and PATDT are based on the physics of heat transfer; PPT performs signal processing in the frequency domain, while SPCT and ICT are unsupervised machine-learning methods capable of dimensionality reduction and feature extraction. Most recently, Liu et al. [4] showed that factor analysis thermography (FAT) achieved similar results to SPCT, while image segmentation based on fuzzy c-means clustering further improved the results. Figure 4 displays the processing results of the thermograms collected in the heating stage, showing that all three methods identified defects A, B, and C in the *Madonna* panel painting. Defect D, the deepest among all four defects, was not revealed by any of the above methods, reflecting an inherent limitation of PT which is usually suited to detect relatively shallow defects.

**Figure 5:**
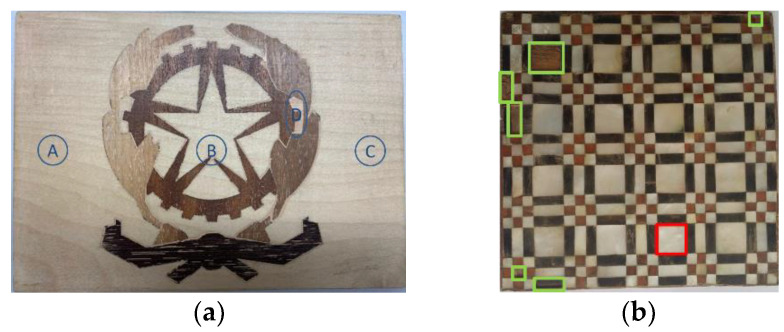
Marquetry samples: (**a**) a panel representing arms’ coats and (**b**) an ancient sample. The Figures were redrawn considering Refs. [43,58].

**Figure 6:**
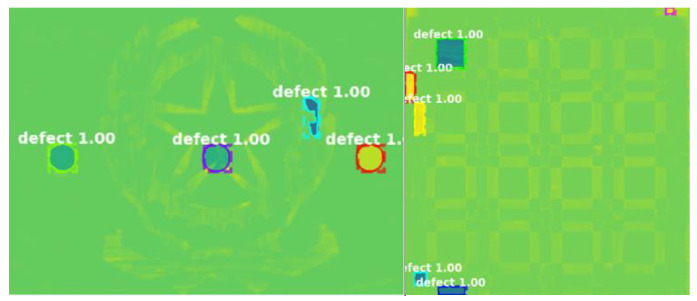
Segmentation results of pulsed thermograms of marquetry samples using Mask R-CNN (Reprinted with permission from Ref. [58]. Copyright: 2021 Garrido, I.; Erazo-Aux, J.; Lagüela, S.; Sfarra, S.; Ibarra-Castanedo, C.; Pivarčiová, E.; Gargiulo, G.; Maldague, X.; Arias, P.).

According to Merriam-Webster, marquetry is a “decorative work in which elaborate patterns are formed by the insertion of pieces of material (such as wood, shell, or ivory) into a wood veneer that is then applied to a surface (as of a piece of furniture)”. In [56], Hu et al. presented a detailed inspection of marquetry panels representing coats of arms. The middle layer of each sample is made of medium-density fiberboard, while the decorative coatings are constituted of different types of woods, including maple, mahogany, maple, wenge, and walnut. One of the samples is defect-free, while the other contains three subsurface flaws (A, B, C) and one superficial putty insert (D), as illustrated in Figure 5a. On the same samples, a number of nondestructive testing techniques were used in [43], including square pulsed thermography. Numerical simulations were conducted to estimate the optimum heat flux to be provided on the front side of the samples. After obtaining the thermograms, the physics-based thermographic signal reconstruction method and the data-driven principal component thermography (PCT) method were adopted as the post-processing methods to enhance the detection results. Another example found in the literature is an ancient marquetry sample, which is a composite of several different types of materials. The support board of this sample is made of fir, while the composition of the decorative layer includes nacre, bovine horn, and boxwood. The locations of defects are shown in Figure 5b, where the green boxes indicate the loss of surface decorations and the red box is a delamination defect. In [57], Wen et al. developed an edge-group sparse PCT (ESPCT) method for the thermographic data analysis of the thermograms of this sample collected in a PT experiment. ESPCT can be regarded as an extension of PCT by imposing sparsity and spatial connectivity constraints. For defect detection in the ancient marquetry sample, ESPCT outperforms PCT and SPCT. Another investigation of both types of marquetry samples was presented in [58]. In this work, pre-processing algorithms were conducted to compensate for the non-uniform backgrounds contained in the thermograms and highlight the most significant thermal footprints; then, a deep learning model named mask region-convolution neural network (Mask R-CNN) was trained for defect detection and segmentation. Figure 6 visualizes the results obtained by the deep learning model. Different from the methods used in [43,57], Mask R-CNN belongs to the family of supervised learning, which means a labeling step is necessary before the model training. In addition, the generalization performance of this model to other marquetry samples has not yet been tested. Nevertheless, this work may be the first attempt to inspect thermographic data of cultural heritage with deep learning models.

### 2.3. Mosaics

Non-destructive testing and evaluation (NDT&E) techniques, such as thermal imaging (IRT), ground penetrating radar (GPR), digital microscopy (DM), microwave reflectometry (MWR), electrical resistivity tomography (ERT), seismic methods (sonic and ultrasonic tests) are widely used in the field of cultural heritage construction due to their non-contact nature, their rapid and simple implementation, their suitability for large-scale inspections and complex surface monitoring [59,60,61,62,63,64,65,66]. Several research works have been conducted applying various thermographic approaches (active and passive), GPR, DM, and seismic methods for the assessment and evaluation of monuments and historical structures, such as mosaic artifacts [61,62,63,64,65,66,67,68,69]. There are also non-invasive methods that have been applied in laboratory conditions, such as electro-optic holography (ESPI), digital holographic speckle pattern interferometry (DHSPI) applied in combination with stimulated infrared thermography (DHSPI-SIRT), holographic subsurface radar (HSR), scanning laser doppler vibrometry (SLDV) [59,60,64].

Mosaics constitute one of the most interesting decorative legacies of antiquity and multilayered structural complexes subjected to various conservation and restoration interventions [61,62,63,67,68,69,70]. Meaningful diagnosis is required for the comprehensive characterization, interpretation, and rating of mosaics’ condition. On the other hand, the identification and characterization of different damage modes affecting these monuments are also of fundamental importance. According to the strict regulation where sampling is restricted and is only limited in some cases, the need for the development and application of nondestructive methodologies is of utmost importance.

Passive and active thermography have been employed as diagnostic tools in the conservation field for the non-destructive identification of hidden defects and internal structural damage of mosaics [69,70,71]. Although passive thermography has been applied as a standard quality control technique for a long time, the thermographic inspection during and after a heating process has been proven to increase the potentiality in the absence of natural thermal contrasts [71,72,73]. Variations in the thermal properties in defective multilayer materials produce thermal effects, which are easily observable via active thermography (i.e., after the application of heat flows into the material bulk) [74,75]. A user-friendly procedure based on an active approach, targeting a long-term and sustainable approach for preserving ancient mosaic heritage was proposed by Theodorakeas et al. [76,77]. Active thermography was used in the lab to test a plastered mosaic in order to reveal the hidden artifact and gain information regarding its location, its thickness, and its characterization through its thermal properties. Quantitative information about the hidden artifact was retrieved through the simultaneous monitoring of the surface temperature decay over a reference (just plaster) and a mosaic-consisted area, and through the conduction of numerical parametric studies for the evaluation of specific parameters alterations (i.e., covering thickness, mosaic layer thickness, thermophysical mosaic properties) on the thermal response of the structure [76,77].

Furthermore, GPR has been established as a powerful diagnostic tool in the field of mosaics’ investigation, capable of providing valuable information regarding the decay and preservation state. Several research works have been published in the literature demonstrating the application of GRP technique for the detection and assessment of subsurface voids and discontinuities on mosaics [24,25,26,61,62,63], the location of doubtful zones, including delamination, changes of structure (recent repairs), or buried heterogeneities and monitoring of mosaic grouting [76]. In addition, a combined methodology including GPR, IRT, and fiber optic microscopy (FOM) has been applied to the mosaics of *Hagia Sofia* in order to assess internal damage and evaluate the performance of previous conservation/restoration interventions, as well as to verify the presence of mosaics in layers below external plastered surfaces [61,62].

**Figure 7:**
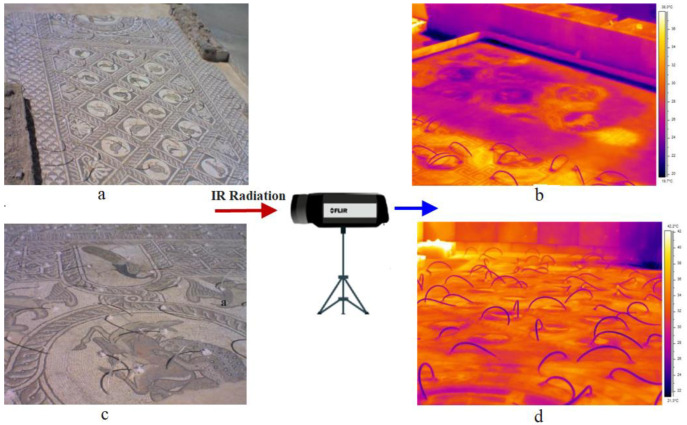
(**a**) Panoramic view of the Delphi floor mosaic’s eastern area, (**b**) representative thermal image without a drainage system, (**c**) mosaic area of animal representations on the tessellated layer, and (**d**) representative thermal image with the drainage system. (Reprinted under the terms of the Creative Commons Attribution 3.0 license from Ref. [77]).

The capabilities of passive and active thermography for the inspection of mosaic structures have been well documented. In situ thermography was applied to examine three mosaic pavements [77]. The first mosaic tested was a pavement consisting of stone-glass tesserae, discovered at the archaeological site of Delphi and dating back to the Early Byzantine Period (5th century A.D). Visual observations revealed local regions with detached tesserae, while the climate conditions where the mosaic is located, are also harsh during the winter time (Figure 7).

The above mosaic’s thermographic investigation was performed at noon when the solar energy was directly heating the inspected structure, producing a transient thermal regime. Representative thermal images from the inspection of the Delphi mosaic are illustrated in Figure 7b,d, where temperature variations were observed on the decorative surface layer. The comparison of the two representative thermographs from an area with and without a drainage system indicated that deterioration phenomena are more aggressive along the eastern area of the mosaic, where the water accumulation occurred due to the lack of a drainage system [77].

**Figure 8:**
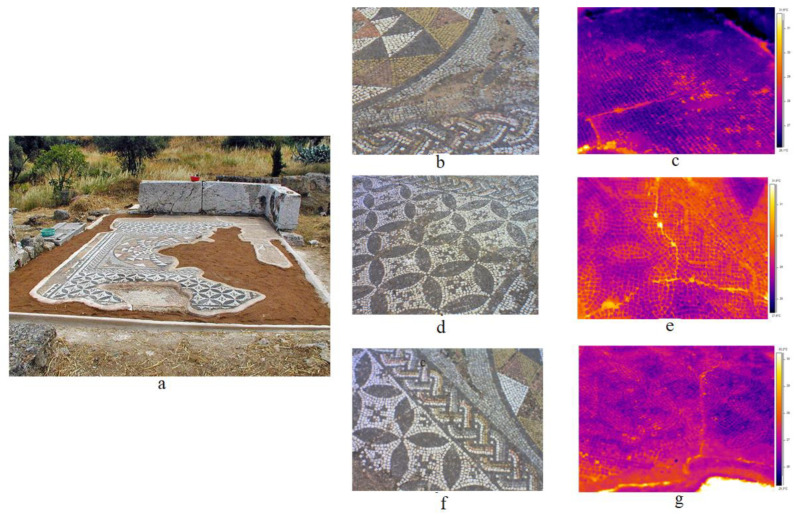
(**a**) Mosaic pavement from the ruins of an ancient house located at the Ancient Agora of Athens, (**b**) a region with surface cracks, (**c**) representative thermal image with visible cracks, (**d**,**f**) regions without surface cracks, (**e**,**g**) representative thermal image with invisible surface cracks. (Reprinted under the terms of the Creative Commons Attribution 3.0 license from Ref. [77]).

Passive IRT was also employed in the investigation of the mosaic floor in the ruins of an ancient house at the Ancient Agora of Athens. The mosaic floor dates back to the second century B.C. and is decorated with geometric patterns and the famous Roman design of parrots drinking water (stone and glass tesserae). Apart from the visible surface cracks, thermographic inspection revealed some further cracking, which was either difficult to be detected through visual testing or undetectable in some instances (Figure 8). Due to the incidental detection of cracks, this phenomenon is probably attributed to the conservation procedure performed by placing cement mortar as a substrate layer, enhancing the incompatibility of the structure in terms of materials’ lay-up and mechanical behavior [24].

**Figure 9:**
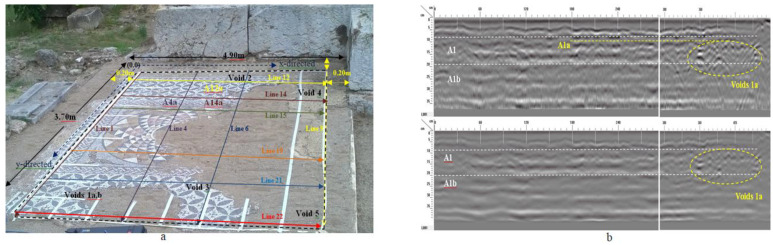
(**a**) The 2D radar data acquisition along single profile lines in the x- and y- directions of the mosaic floor, (**b**) The 2D radar profile acquired along the y- directed line 1, time zero, and migrated profile. (Reprinted with permission from Ref. [24]. Copyright: 2017 Ftikou, Ε.; Theodorakeas, P.; Cheilakou, Ε.; Koui, Μ.).

Along with the thermographic inspection and in order to gain knowledge on the structural integrity of the infrastructure, deeper probing was applied through the aid of GPR. Reflections were observed to a depth of 0.05 m from the surface, revealing the presence of a layer with different dielectric properties compared to the background (Figure 9). This target corresponds to the tessellated layer of the Hellenistic mosaic, which represents the first constructive phase of the monument, subsequently covered by the Roman mosaic. The above interpretation was verified by the archeological/conservation report of the historic landmark, where the depth of the hidden Hellenistic mosaic was in a depth ranging from 0.05 to 0.07 m from the surface. Further reflections were also detected, which can be related to the substrate of the respective mosaic consisting of coarse materials. Due to their undefined shape and orientation, they are probably related to weather material and/or water-filled voids (Figure 9b).

**Figure 10:**
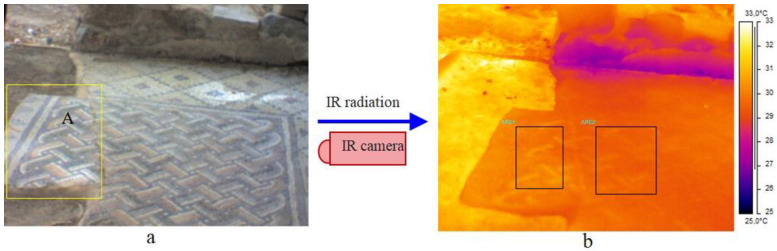
(**a**) View of the mosaic pavement located at the “Sanctuary of Pan”, which is situated on the Pnyx slopes, northwest of Acropolis in Athens, (**b**) representative thermal image. (Reprinted under the terms of the Creative Commons Attribution 3.0 license from Ref. [77]).

The archeological site of Sanctuary of Pan was discovered in 2001 and dated between the second century B.C. and first century A.D. It includes, outside the caved chamber, a rectangular room with a floor mosaic of geometric patterns. The visual inspection demonstrated that the mosaic structure was in a good state of preservation. This observation was also confirmed through the use of thermographic testing (Figure 10). As it is presented in Figure 10b, the surface temperature uniformity was detected on the decorative layer of the mosaic. Nevertheless, the humidity was detected at the consolidated edges mainly next to the lateral cave wall, even if a tent was placed vertically on the cave wall. Additionally, the thermal compatibility of new (AR01) and ancient (AR02) (in which AR01 and AR02 stand for area1 and area2 in Figure 10, to the right) mortar was confirmed [24,25,26].

**Figure 11:**
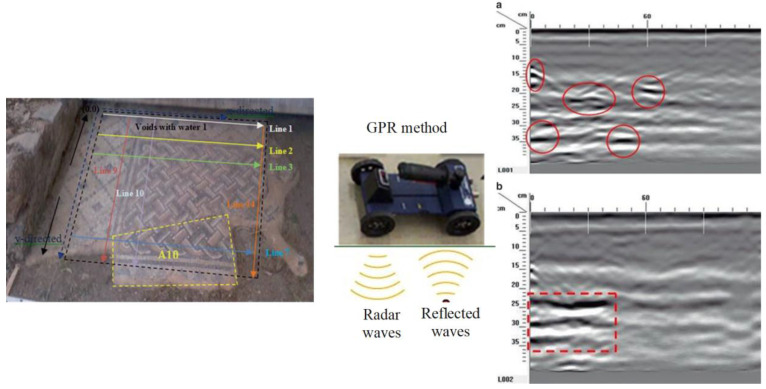
The 2D radar acquisition along single profile lines in the x and y directions of the mosaic (**left**), 2D radar profiles acquired along *x*- direction line 1 (**a**) and line (**b**) (**right**). Discontinuities in the medium over a depth of 0.10 to 0.35 m are highlighted with red lines. (Reprinted with permission from Ref. [24]. Copyright: 2017 Ftikou, Ε.; Theodorakeas, P.; Cheilakou, Ε.; Koui, Μ.).

As this measurement refers only to the first few centimeters below the decoration, the examination was combined with the GPR technique to identify shallow voids and discontinuities, the location of doubtful zones, including delamination, changes of structure (recent repairs), or buried heterogeneities, and for monitoring of mosaic grouting [24,25,26]. Data collected from the 2D profiles of the GPR network scattering within the mosaic pavement revealed subsurface distortions in the mosaic’s substrate that are probably related to weathering of the material and/or water-filled voids (Figure 11).

**Figure 12:**
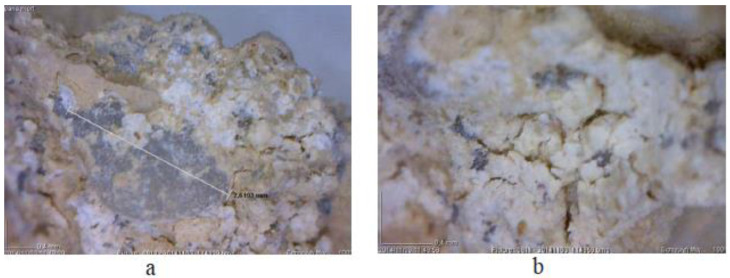
(**a**) Microphotographs of the mortar sample show the presence of pozzolan tracers and gray grains of aggregates, the length of which reached 2.61 mm, (**b**) a thin off-white layer corresponding to the carbonate matrix. (Reprinted with permission from Ref. [24]. Copyright: 2017 Ftikou, Ε.; Theodorakeas, P.; Cheilakou, Ε.; Koui, Μ.).

The observations derived from the examination of the mortar specimens by means of DM revealed that the mosaic layer structure is “opus tessellatum” type. This structure consists of three layers of mortars and one infrastructure layer containing coarse aggregates. The thickness of the layers, as well as the aggregate sizes, decreases toward the upper layers, while the ratio of bonding slurry to aggregates increases. DM examination revealed the presence of a carbonate matrix as well as the presence of pozzolan tracers or brick fragments in the mortar (Figure 12). Thus, it is suggested that this is a pozzolanic mortar [26].

## 3. Conclusions

In this work, the applications of pulsed infrared thermography (PT) [78] to the analysis of three different types of Cultural Heritage artifacts, namely documentary materials, panel paintings, marquetery, and mosaics, have been reviewed. In the first application, PT was used for the detection of graphic features in library/archive items, such as texts on scraps buried within ancient bindings which may contain valuable information for restorers and scholars. The use of such a technique in combination with MWIR Reflectography also enabled the possibility of distinguishing between inks characterized by different compositions, thus providing valuable indications for this kind of investigation. After that, studies reporting PT investigations of panel paintings and marquetery were presented. In particular, the image processing tools recently introduced to enable the visualization of otherwise undetectable features were discussed. Finally, the use of PT in combination with passive infrared thermography for the analysis of mosaics was extended [79]. Such an approach allowed the inspection of large areas thanks to the heating provided by the sunlight. In addition, chemical–physical techniques have also been employed in order to detect shallow features. The results verified the effectiveness of the combined applied methodology of IRT, GPR, and DM for the inspection of mosaic artifacts, offering key advantages such as instrumentation mobility, fast data acquisition, large-scale inspections, real-time interpretation of the collected data, contributing significantly to their conservation—restoration procedures. For each of the above-mentioned PT applications, the limitations and the latest actions taken to overcome them were also discussed, providing an overview of the various research activities currently underway to further improve PT effectiveness [80].

## Figures and Tables

**Table 1:** Reasons for the thermographic inspection of the selected cultural heritage objects.

	Feature Inspected	Structure for Scientific Reasons	Identifying Hidden Features (e.g., Underdrawings, Pentimenti, Preparation Layers by Pencil)	Identifying Defects for Subsequent Treatment and Restoration	Identifying Forgery	Identifying Damaged Features
Artwork						
Documentary materials		*√*	*√*	*√*		*√*
Panel paintings		*√*	*√*	*√*	*√*	*√*
Marqueterie		*√*		*√*		*√*
Mosaics		*√*		*√*		*√*

## Data Availability

Not applicable because this is a review article.
